# Bovine Neonatal Monocytes Display Phenotypic Differences Compared With Adults After Challenge With the Infectious Abortifacient Agent *Neospora caninum*

**DOI:** 10.3389/fimmu.2018.03011

**Published:** 2018-12-19

**Authors:** Parul Sharma, Catherine S. Hartley, Manjurul Haque, Tracey J. Coffey, Sharon A. Egan, Robin J. Flynn

**Affiliations:** ^1^Department of Infection Biology, Institute of Infection and Global Health, University of Liverpool, Liverpool, United Kingdom; ^2^School of Veterinary Medicine and Science, University of Nottingham, Nottingham, United Kingdom; ^3^Institute of Parasitology, McGill University, Sainte Anne de Bellevue, QC, Canada

**Keywords:** neonatal, monocyte, *Neospora canium*, immunity, JAK-STAT, bovine

## Abstract

The neonatal period represents a window of susceptibility for ruminants given the abundance of infectious challenges in their environment. Maternal transfer of immunity does not occur *in utero* but post-parturition, however this does not compensate for potential deficits in the cellular compartment. Here we present a cellular and transcriptomic study to investigate if there is an age-related difference in the monocyte response in cattle during intra-cellular protozoan infection. We utilized *Neospora caninum*, an obligate intracellular protozoan parasite that causes abortion and negative economic impacts in cattle worldwide, to study these responses. We found neonatal animals had a significant greater percentage of CD14^+^ monocytes with higher CD80 cell surface expression. Adult monocytes harbored more parasites compared to neonatal monocytes; additionally greater secretion of IL-1β was observed in neonates. Microarray analysis revealed neonates have 535 genes significantly upregulated compared to adult with 23 upregulated genes. Biological pathways involved in immune response were evaluated and both age groups showed changes in the upregulation of tyrosine phosphorylation of STAT protein and JAK-STAT cascade pathways. However, the extent to which these pathways were upregulated in neonates was much greater. Our findings suggest that neonates are more resistant to cellular invasion with protozoan parasites and that the magnitude of the responses is related to significant changes in the JAK-STAT network.

## Introduction

Neonatal mortality amongst production livestock remains an endemic problem within developed and developing countries. Environmental “insults,” including infectious challenges, are present from birth, and together with a lack of a fully educated adaptive compartment poses a particular challenge to neonatal survival placing pressure on food production systems and food security.

The development of the ruminant immune system would suggest that colostrum offers immediate protection by way of antibody transfer, while the adaptive compartment develops an incrementally protective clonal response (1, 2). There is some work which challenges this paradigm as Reber et al. (3) have demonstrated a transfer of maternal leukocytes into neonatal circulation, the maternal cell compartment contributed 3% of the total circulating neonatal pool. However, these cells are no longer detectable by 36 h post transfer. This paradigm for incrementally increasing adaptive clonal responses is well illustrated by the dynamics of nematode challenge—whereby hosts exposed on the first occasion develop large nematode worm burdens while adults with repeated exposures harbor lower nematode numbers (4, 5) due to the efficacy of the clonal populations in their adaptive compartments. This paradigm is challenged by the enhanced resistance of calves to *Babesia bovis*, whereby calves display lower parasitemia, clinical disease, and greater inflammatory reactions. These responses included enhanced nitric oxide (NO) and interleukin (IL)-12 production which were all dependent on the presence of the spleen and NK-like cells (6, 7). NK cells could potentially be important effectors during this phase as they are not reliant on antigen specificity. Broadly, a heightened innate immune response would offer wide protection against multiple pathogen types due to the use of pathogen recognition receptors (PRR) across the innate system, avoiding reliance on a fully-antigen experienced adaptive response. Moreover, within the innate system the same cell can act as both pathogen sensor and effector resulting in rapid responses to pathogens. Therefore, given the absence of a full antigen-experienced adaptive system during this early life period we sought to extend the findings above and sought to define a potential mechanism by which the neonatal immune system can compensate for the absence of fully functional adaptive response.

*Neospora caninum* is a protozoan abortifacient agent that causes major economic and welfare losses in the cattle industry. It is capable of both horizontal and vertical transmission within herds and pregnant animals, respectively (8). Moreover, placental material derived from infected heifers can serve as a source of infection as can oocysts in the environment (9). Live vaccination can prevent abortion, but causes life-long infection making protective vaccination a research priority for calves and adults (10). Here we demonstrate that while neonatal monocytes contain lower numbers of *N. caninum* compared to adults following *in vitro* challenge they are more sensitive in terms of inflammatory reactions. They secrete greater amounts of IL1β and IL-6 and trigger enhanced NK cell activation downstream. Microarray analysis of monocytes reveal a higher inflammatory baseline in neonates and greater activation of the JAK-STAT and tyrosine phosphorylation of STAT pathways upon infection in neonates when compared with adults.

## Materials and Methods

### Animals

Holstein X Friesian (HxF) male calves 10 days of age were acquired from the University of Nottingham Dairy Unit, and blood was collected into lithium heparin, following schedule 1 culling of animals by captive bolt. Blood from adult HxF, between 18 and 24 months was acquired at commercial abattoirs in Derbyshire and Greater Manchester. All animals were clinically healthy at the time of sampling and negative for *Cryptosporidium parvum*, bovine respiratory disease, and *Neospora caninum*. At the time of sample collection no pathological indications of disease were reported.

### Ethics

All procedures were reviewed and approved by University of Nottingham AWERB prior to collection of samples. Animals involved had their suffering minimized and their welfare was maintained as per standard husbandry.

### Parasite Culture

The *Neospora caninum* isolate NcLiv was used throughout and cultured as previously described (11) with the addition that parasites were stained with CFSE prior to use. Parasites were stained using 5 μM CFSE/10^7^ parasites for 10 mins at RT. At the end of incubation period 400 μl of RPMI complete media was added and parasites kept on ice for 5 mins then washed at 448x *g* for 5 mins. The supernatant was aspirated and parasites were resuspended in 1 ml of D-PBS for further use.

### Monocyte and NK Cell Culture

Buffy coats were prepared from animals and used to positively select CD14^+^ monocytes as previously described (11). In separate experiments the depleted fraction was used to isolate CD335^+^ NK cells in a two-step fashion with a primary anti-CD335 (Clone AKS1 rat IgG1) incubation followed by anti-rat IgG microbeads. All cells were cultured in completed RMPI, 10% FCS, 100 U/mL penicillin, and 100 μg/mL streptomycin. CD14 monocytes were seeded at 2 × 10^5^ cells/well and infected with 1 × 10^5^ purified stained *N. caninum* tachyzoites for 24 h. NK-cells were counted and seeded into 24 well plates at 1 × 10^4^ cells/well with overnight stimulation with recombinant bovine IL-15 (Kingfisher Biotech Inc. 10 ng/ml). NK-cells were then added to autologous monocyte cultures for a further 24 h at a ratio of 1:10.

### Flow Cytometry Analysis

Leukocytes were prepared by washing of 1 × 10^7^ cells in fresh RPMI complete media and resuspending FACS buffer (10% FBS in D-PBS). Cells were incubated with Fc block (0.5% BSA in D-PBS). Staining was performed with mouse anti-bovine CD14 (Clone CC-G33, FITC, IgG1); CD80 (Clone IL-A1259, RPE, IgG1); CD86 (Clone ILA190, RPE, IgG1); MHCII (Clone IL-A21, RPE, IgG2a). Matched isotypes were used throughout.

Cells were kept on ice protected from light until flow cytometry was performed on a BD FACS Canto-II. Cells were gated on the basis of forward scatter (FSC) and side scatter (SSC) parameters to exclude dead cells or debris. Unstained cells and isotype controls were used to set gating thresholds. For dual staining i.e., FITC and RPE, appropriate compensation settings were applied as necessary at the time of sample acquisition. A minimum of 10,000–30,000 events were recorded per sample.

### Cytokine Detection

ELISAs were performed to measure bovine IL-1β and IL-6 (Catalog no. ESS0027 and ESS0029, respectively Thermofisher Scientific, UK) according to manufacturer's instructions.

### Microarray

RNA isolation was performed from infected and uninfected CD14+ monocytes grown in a monolayer using an RNAeasy kit. Samples were subject to DNase removal by an Ambion DNase DNA free kit. Microarray was performed using an Agilent One-Color The Bovine (V2) Gene Expression Microarray, 4 × 44K chip (G2519F-023647). Differential expression and changes in network representation was analyzed using NetworkAnalyst (12).

### Statistical Analysis

All data were collected in EXCEL and analyzed in Graphpad Prism V7.0; specific details of tests used are given in the figure legends and a *P* < 0.05 was taken as significant.

## Results

### The Circulating Monocyte Population Differs Amongst Neonates and Adults

**Figure 1:**
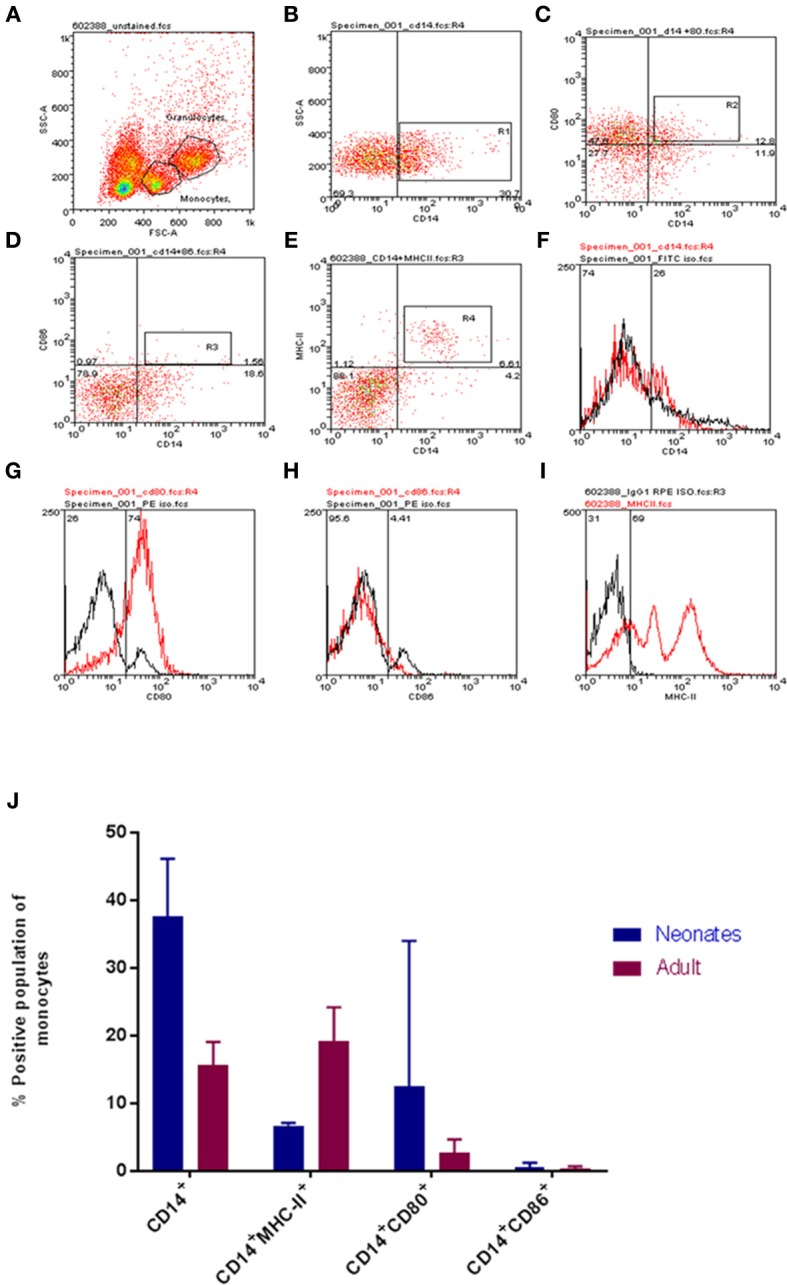
Age-dependent differences in bovine monocyte proportions and phenotypes. Representative gating for neonatal and adult leukocytes is shown. Cells were identified on FSC vs. SSC dot plot **(A)**, then CD14 positive cells **(B)**, CD14CD80 dual staining **(C)**, CD14 CD86 dual staining **(D)**, CD14 MHCII dual staining **(E)**, isotype controls for CD14, CD80, CD86, and MHCII are shown in **(F–I)**. **(J)** The proportions of defined cell populations are presented for both age groups. Cell populations presented are CD14^+^; CD14^+^MHC-II^+^; CD14^+^CD80^+^; and CD14^+^CD86^+^. Our analysis revealed a larger total population of CD14^+^ monocytes and CD14^+^CD80^+^ expressing monocytes within calves; while adults harbored a larger population of CD14^+^MHC-II^+^ monocytes. The bars show mean ± SEM of adult and young samples (*N* = 3 and 4 per group, respectively). Results were analyzed using GraphPad Prism 7.02 and statistical analysis was performed out by Mann Whitney test to compare the means of defined populations between age groups; *indicates a *P*-value < 0.05.

To begin our investigation the monocyte pool was examined for differences between neonates and adults. After initially establishing a forward scatter (FSC) and side scatter (SSC) gate, a pool of CD14^+^ monocytes were identified (Figure 1A). The total CD14^+^ monocyte population was determined as were dual staining populations of CD14^+^CD86^+^ and CD14^+^CD80^+^ cell populations (Figures 1A–J for neonates and Figure S1 for adults). Our results indicated that neonates had a greater total CD14^+^ monocyte pool (*P* < 0.05; Figure 1J) and that within this the CD80 dual positive pool was also larger (Figure 1J). Neither young nor adult CD14+ monocytes showed detectable expression of CD86 within the CD14^+^ population; significant individual to individual variation was noted (Figure 1J). MHC-II levels were determined to be significantly different as adult monocytes displayed higher levels of MHC-II compared to their neonatal counterparts (*P-value* = *0.005*, Figure 1J).

### *Neospora caninum* Challenge Reveals a Functional Difference in Neonatal and Adult Bovine Immune Responses

**Figure 2:**
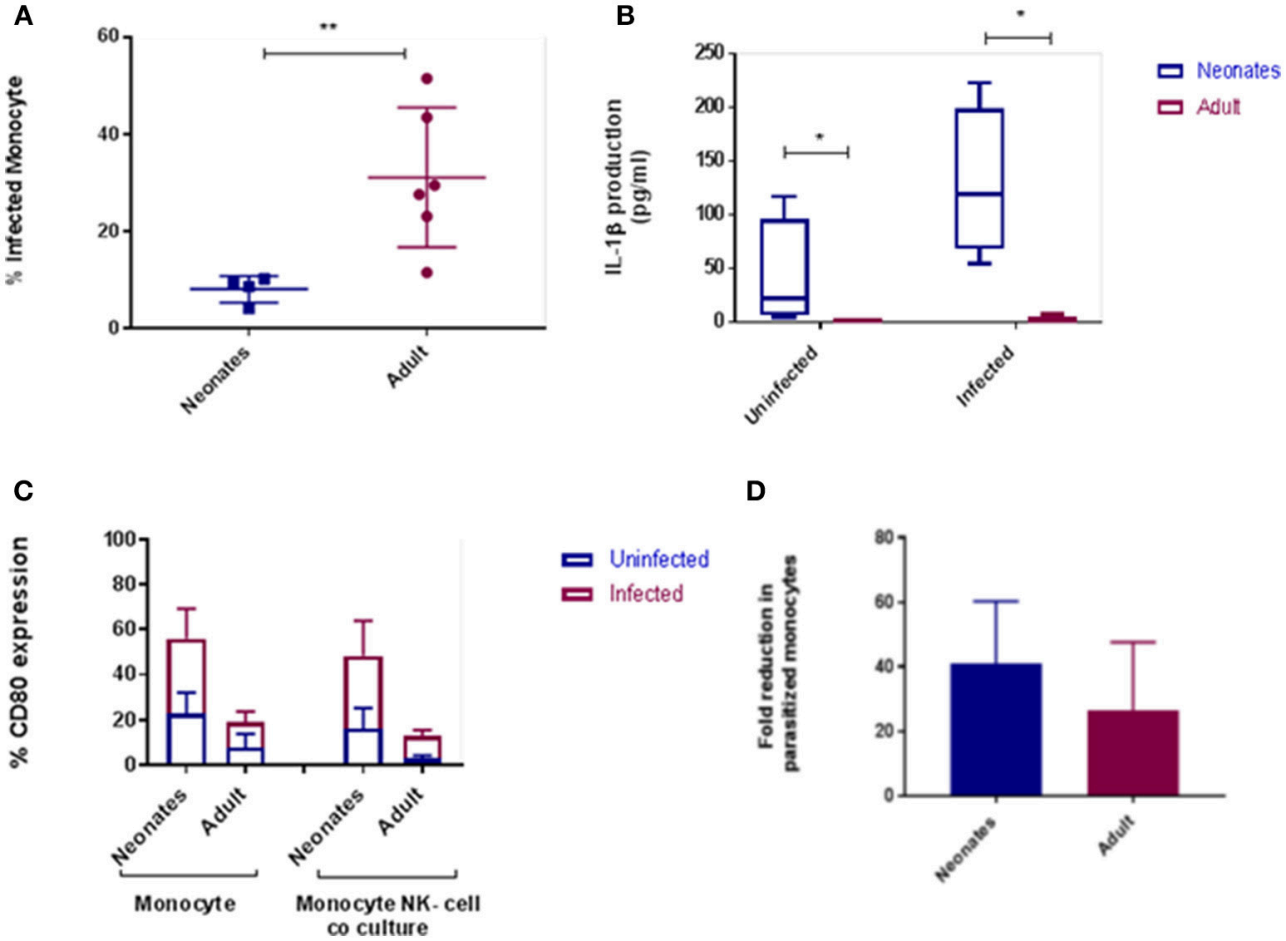
Cattle monocytes were infected with CFSE labeled *N. caninum* tachyzoites. **(A)** Parasite uptake was determined by flow cytometry 24 h post infection. These result shows significant difference between the percentage of infected neonates (*N* = 4) and adult (*N* = 6) CD14^+^ monocytes *P*-value < 0.01. **(B)** IL-1β production was in supernatants by ELISA after infection. The significance difference was noticed between neonate and adult infected and uninfected monocytes, *P*-value < 0.05. Data shows median with 95% confidence intervals, result were analyzed using Prism Graphpad 7.02 and statistical analysis was performed by using Mann Whitney test. **(C)** CD80 expression of monocyte and monocyte-NK cell co-cultured cells (10:1) from *N. caninum* infected and uninfected control samples after 24 h. Result were analyzed using GraphPad Prism 7.02 and statistical analysis was performed by 2 way ANOVA with Sidak's multiple comparison to determine the significance difference between neonates and adult. **(D)** Parasitized CD14^+^ monocytes co-cultured with autologous NK-cells were examined for parasite load after 24 h as above. Results, means plus 95% C.I., are presented as fold reduction compared with baseline parasite load pre-co-culture. *indicates a *P*-value < 0.05 and **indicates a *P*-value < 0.01.

To extend our findings to an infectious challenge we labeled tachyzoites of the parasite *N. caninum* with carboxyfluorescein succinimidyl ester [(CFSE); Nc-CFSE] and challenged CD14^+^ monocytes with Nc-CFSE at a multiplicity of infection (MOI) of 4 for 24 h and subsequently infected cells were identified using FACs analysis. This allowed for an accurate estimation of proportions of cells infection (Figures S1, S2). Analysis of neonatal and adult responses demonstrated that the rate of infection was lower in neonatal monocytes (Figure 2A). In tandem with this we also found that IL-1β secretion by infected neonatal monocytes was significantly higher (*P* < 0.05; Figure 2B), IL-6 secretion was also higher, but this was not significantly different (Figure S3). We assessed if expression of cellular CD80 was upregulated in this context, and found that after *N. caninum* challenge neonatal monocytes displayed greater levels of CD80 expression relative to their adult counterparts (Figure 2C). To test the functional significance of this, we co-cultured infected monocytes with autologous CD335^+^ NK cells, ratio of 10:1, for a further 24 h. Thereafter we determined the fold reduction in Nc-CFSE and found that neonatal monocytes underwent a 40% reduction in parasite burden compared with adult monocytes achieving a reduction of 25% (Figure 2D).

### Neonatal Monocytes Differ From Adult Monocytes in Their Gene Network Activation

**Figure 3:**
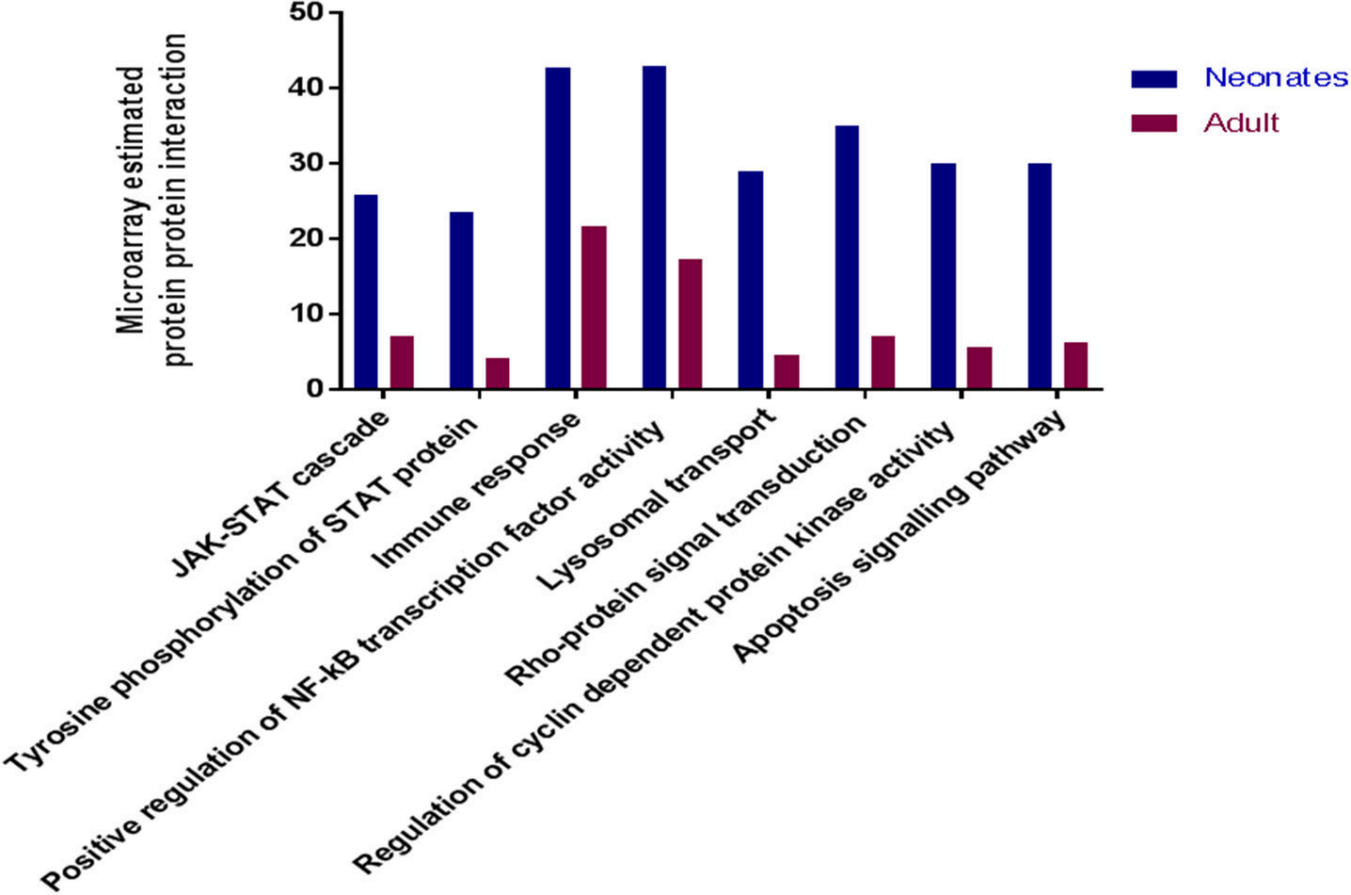
Microarray analysis of infected monocytes show neonatal vs. adult differences. Common immune related KEGG pathways which were upregulated in neonatal and adult cattle monocytes infected with *N. caninum* vs. uninfected monocytes. These bars represent the percentage of estimated protein-protein interactions in common biological immune responses pathway of neonates (*n* = 3) and adult (*n* = 2) cattle.

**Figure 4:**
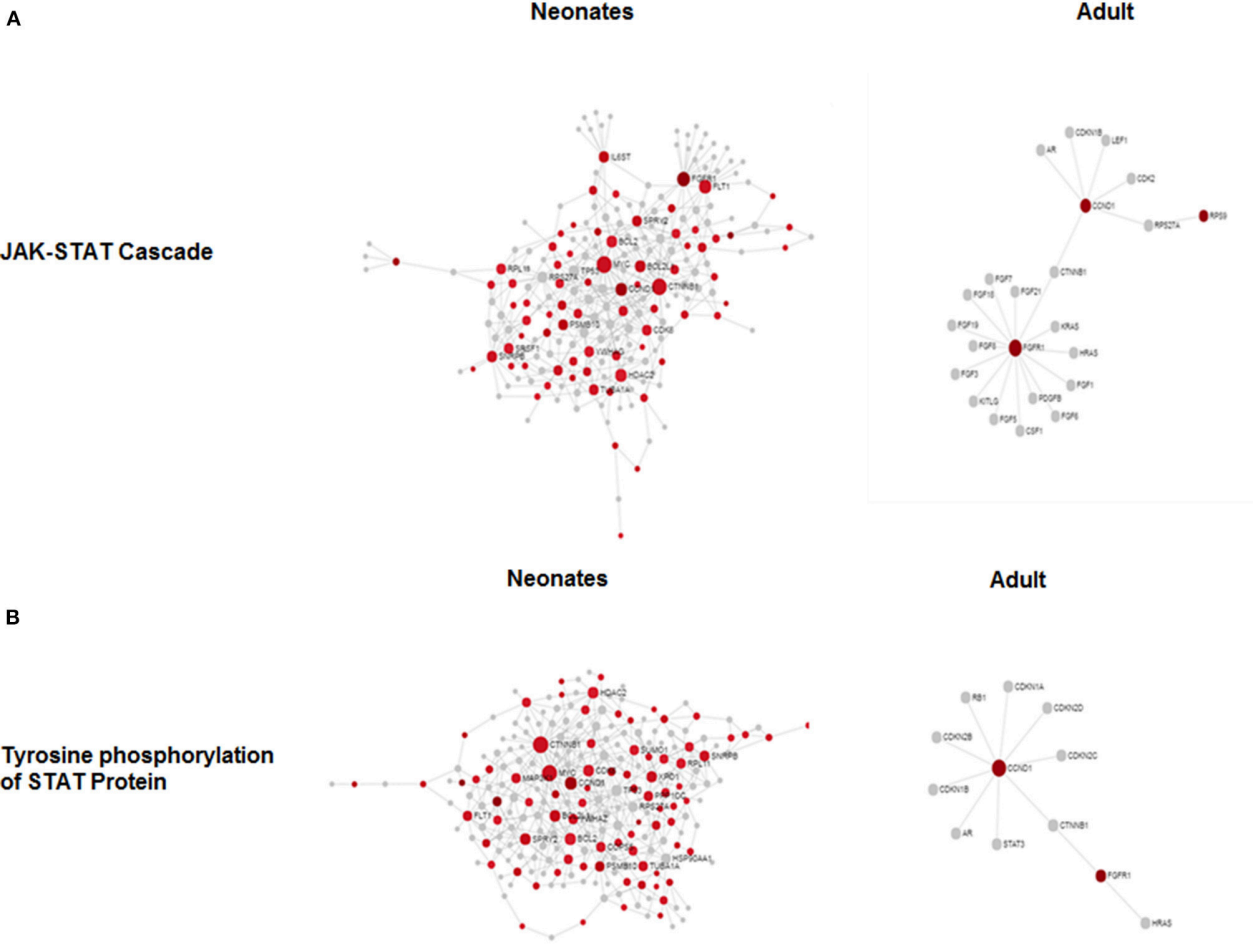
Microarray network perturbation in neonates and adult monocytes following *N. caninum* challenge. Changes in **(A)** the JAK-STAT and **(B)** tyrosine phosphorylation of STAT networks, as analyzed by microarray, were visualized by network diagrams (networkanalyst.ca) where red nodes represent the upregulation of genes during protein-protein interaction while gray nodes represent no expression of genes in corresponding pathway. Both pathways show extensive upregulation of genes in both networks in neonatal in comparison to adults.

To determine if there were global differences in neonatal monocytes compared with adult monocytes, we challenged cells as above and processed them for microarray analysis. An initial analysis, using a >2-fold change cut-off, identified 523 differentially expressed genes in neonatal cells compared with 23 differentially expressed genes in adult cells. The top 10 upregulated transcripts for both neonates and adults are shown in Table 1, with only one gene shared between the two groups post-infection, ribosomal protein L10 like (RPL10L). The top 3 genes uniquely upregulated in neonates were all involved in the immune response compared to the adult samples. A network analysis (12) found 120 altered pathways in neonates compared with 53 in adults (Supplementary Data and Supplementary Tables). Of these pathways, 25 neonatal pathways were directly involved in the immune response compared with only 10 in the adult samples. A side-by-side comparison of these revealed stark differences in the predicted protein-protein interactions (Figure 3). We estimated there was a range of 20–40% protein-protein interactions amongst these pathways within neonates including the JAK-STAT, tyrosine phosphorylation of STAT, and positive regulation of NF-κB pathways, this compared with a range of 5–20% for adults. When we further visualized the JAK-STAT (Figure 4A) and tyrosine phosphorylation (Figure 4B) of STAT pathways it was clear that these networks were more extensively activated with a wider degree of interactions in the neonates compared to their adult counterparts. Upregulated nodes, indicated in red, included *socs2, il6st*, and *fgfr1* (Figure 4B) and *sumo1* and *map3k1* (Figure 4B). STATs in particular have been implicated widely in intracellular protozoan immunity and differential activation appeared to be essential to the age-dependent differences evident in our system.

**Table 1:** Top 10 differentially expressed genes in neonatal and adult monocytes.

**Neonates**			**Adult**		
***Gene name***	**Fold Expression**	***P*-value**	***Gene name***	**Fold Expression**	***P*-value**
*ICAM3* Intercellular adhesion molecule 3	7.1	0.003	*RPL10L* Ribosomal protein L10 like	7.14	0.001
*CCL2* C-C motif chemokine ligand 2	5.0	0.003	*PFN2* Profilin 2	5.65	0.024
*TMED3* Transmembrane p24 trafficking protein 3	4.74	0.003	*TOMM7* Translocase of outer mitochondrial membrane 7		0.024
*CCFC69* Coiled-coil domain containing 69	4.72	0.003	*SFN* Stratifin	5.39	0.024
*SOCS2* Suppressor of cytokine signaling 2	4.70	0.003	*PPIA* Peptidyl-prolyl cis-trans isomerase A Peptidyl-prolyl cis-trans isomerase A, N-terminally processed	5.32	0.024
*CCDC50* Coiled-coil domain containing 50	4.14	0.003	*FN1* Fibronectin 1	5.32	0.024
*CTTN* Cortactin	4.13	0.007	*UCHL1* Ubiquitin C-terminal hydrolase L1	5.31	0.024
*RPL10L* Ribosomal protein L10 like	4.11	0.007	*S1PR2* Sphingosine-1-phosphate receptor 2	5.30	0.024
*CYP1B1* Cytochrome P450 family 1 subfamily B member 1	4.0	0.008	*PHB* Prohibitin	5.294	0.024
*SMAP2* Small ArfGAP2	3.97	0.008	*FGFR1* Fibroblast growth factor receptor 1	5.293	0.024

*Following microarray analysis of infected vs. uninfected CD14^+^ monocytes, age-specific top 10 genes were identified and are listed in the table. A 2-fold change in gene expression was used as the cut-off for differentially expressed genes, all genes in both age groups are upregulated. Only RPL10L is common to both groups indicating a divergent response to N. caninum infection*.

## Discussion

Herein we demonstrate a difference in the surface phenotype of neonatal monocytes compared to adult cells. This cellular phenotype masks greater changes in the response following infectious challenge both at a protein and gene level. The neonatal circulating leukocyte population contained a higher proportion of CD14^+^ monocytes compared with adults and these in turn had greater surface levels of expression of CD80. This data is consistent with studies in humans where monocytes represented a greater percentage of blood mononuclear cells in infants at term vs. adults (13). Our data is particularly relevant when considered in the context of recent studies that identified bovine monocyte subsets homologous to the human and murine classical, intermediate, and non-classical subsets (14). In future our analysis should define whether subsets of monocytes are preferentially infected or preferentially respond to *N. caninum* challenge and if this is altered by age of host. When challenged with *N. caninum*, neonates displayed lower parasite loads and higher cytokine responses. Microarray analysis of infected adult compared to neonatal monocytes demonstrated that neonates had a greater baseline, in terms of numbers of genes upregulated compared with adults. Moreover, in neonates genes and pathways related to the immune responses were overrepresented compared with adults (Supplementary Data and Supplementary Tables). This is suggestive of a heightened innate response at baseline and in response to challenge in neonates.

Our cytokine data suggests that the profile of production is similar but the difference is in magnitude of expression. We demonstrate here that levels of IL-1β is elevated in neonates compared with adults. Our previous studies have shown that IL-1β is produced by infected fibroblasts but we have not examined the impact of age on this system. However, studies in *T. gondii* have demonstrated before that IL-1β an endpoint of inflammasome activation can directly mediate cellular control of infection. In addition, NLRP1 silencing in human monocytes led to reduced production of IL-1β and greater rates of cellular infection (15). Interestingly, the NLRP3-IL-1β response to *T. gondii* in humans is downregulated during monocyte to macrophage transition (16) suggesting that elements of the inflammasome are context specific. Human studies of monocyte subset production of IL-1β suggests that adults are more capable of IL-1β production across all subsets when compared with term and pre-term infants (13). However, not all studies demonstrate a similar pattern, Nguyen et al. (17) demonstrated that stimulus specific induction of IL1-β in infants compared with adults. This mirrors the results from our experiments, where non-infectious stimuli failed to elicit a differential IL-β response (data not shown) but infectious challenge succeeded in doing so (Supplementary Data and Supplementary Tables).

CD80 interactions with NK cells can regulate *T. gondii* infection and resistance *in vivo* (18). These previous studies revealed that CD80 (B7) expression preceded potent killing of target cells (19). In line with these observations our data showed co-culture with autologous NK cells resulted in an additional and more substantial reduction in parasite load. There is *in vivo* evidence that already implicates NK cells in *N. caninum* infection as they have been shown to secrete IFN-γ in response to the parasite killing infected fibroblasts (20), and increase in circulation following inoculation (21).

Underlying these outward phenotypic changes were dramatic differences in the activation of a number of immune pathways amongst neonates compared with adults. Amongst these are the JAK-STAT and tyrosine phosphorylation of STAT pathways, previously all shown to be important during innate responses against protozoan infection (22, 23). Amongst the genes upregulated in this pathways was *socs2*. Studies in human moDCs found *socs2* was upregulated in response to LPS. Silencing of *socs2*, with siRNA, resulted in greater IL-10 production following LPS stimulation, suggesting that *socs2* was responsible for negatively regulating the anti-inflammatory responses (24). This was supported by the findings that the silencing of *socs2* also promoted hyper-phosphorylation of STAT3, a known suppressor of anti-*T. gondii* innate defenses (25). Importantly, evidence in humans already suggests there are subtle-age shifts in JAK/STAT signaling. Tortorella et al. (26) demonstrated a relative failure of aged neutrophils to phosphorylate JAK2 in response to GM-CSF, while aged T-cells had increased SOCS3 protein levels after IL-12 stimulation giving rise to lower p-STAT4 activation (27). Thus, *soc2* upregulation may suppress STAT3 in neonates providing a signaling environment for enhanced parasite killing.

Our evidence presented here suggests that neonates have increased sensitivity to *N. caninum*, a common protozoan abortifacient via upregulated TLR4. This gives rise to increased cytokine secretion with an underlying difference in the activation of JAK-STAT pathways. Given the role of excessive host inflammation in *N. caninum* driven abortion (28), the role of this heightened immune response in non-pregnant animals should be assessed. However, an over-active innate response in neonates during inflammation can drive a higher rate of mortality (29). Thus, our results may yet reveal either a protective or pathological mechanism for bovine neonates during parasite challenge.

## Author Contributions

PS and CH performed experiments. SE, TC, and RF provided reagents. PS, SE, MH, and RF analyzed data. RF, TC, and SE supervised the project. RF and PS drafted the manuscript. All authors edited the final submission.

### Conflict of Interest Statement

The authors declare that the research was conducted in the absence of any commercial or financial relationships that could be construed as a potential conflict of interest.
