# Supplementary material for: Bovine Neonatal Monocytes Display Phenotypic Differences Compared With Adults After Challenge With the Infectious Abortifacient Agent Neospora caninum
Source: Front Immunol. 2018 Dec 19;9:3011. doi: 10.3389/fimmu.2018.03011 (PMC6305741; doi:10.3389/fimmu.2018.03011)
Supplement: Supplementary file 1 [file Data_Sheet_1.docx]

Supplementary Material

Bovine neonatal monocytes display phenotypic differences compared with adults after challenge with an infectious abortifacient agent.

**Parul Sharma^1,2^, Catherine S Hartley^1^, Tracey J Coffey^2^, Sharon A Egan^2^, Robin J Flynn^1^**

*** Correspondence:** Dr Robin J Flynn, Email: [rjflynn@liverpool.ac.uk](mailto:rjflynn@liverpool.ac.uk)

# Supplementary Data

Supplementary Information Text

**Isolation of peripheral blood mononuclear cells (PBMC) from whole blood**

Buffy coat cells were prepared from the whole blood of both adult and neonates by decanting blood into 50ml tubes and centrifuging at 400 x *g* for 30mins. The buffy coat layer between the plasma and erythrocytes was collected with a Pasteur pipette and placed into fresh tubes. Buffy coats were diluted with an equal amount of sterile Dulbecco’s phosphate buffer saline (D-PBS, Sigma Aldrich, UK) and density gradient centrifugation. To obtain PBMCs, approximately 30ml of diluted buffy coat was overlaid onto 15ml of Histopaque 1077 (1.077g/l Sigma Aldrich, UK) and centrifuged at 400Xg for 30mins with the brake in an offsetting. The layer between the histopaque and the erythrocytes was collected and erythrocytes lysed by addition of 5ml of erythrocyte lysis buffer and mixing of cells for 10mins. Samples were centrifuged at 220 x *g* for 10mins and the supernatant of lysed RBC was discarded. To obtain leukocytes from whole blood of neonatal and adult cattle, the buffy coat layer was washed with cold D-PBS and RBC lysed as above and . repeated 2-3 times for complete lysis of erythrocytes. The final wash was performed with D-PBS and RPMI-1640 complete media (Sigma Aldrich, UK) supplemented with 10% fetal bovine serum (Sigma Aldrich, UK) and 100 U/mL penicillin/ 100µg/ml streptomycin (Sigma Aldrich, UK).

**Magnetic separation of CD14+ monocytes population from PBMC**

Stored PBMCs were used for the separation of CD14+ monocyte/macrophages populations. Cryopreserved PBMCs were removed from liquid nitrogen and thawed at room temperature (RT). The cells were washed in 10ml of warm complete media (RPMI 1640, 10% fetal calf serum and 1% penicillin-streptomycin) and centrifuged at 220 x *g* for 10mins at 22°C. The supernatant was discarded, and cell pellets resuspended in 1ml of fresh complete media and adjust to a concentration of 10^7^cell/ml then washed at 300 x *g* for 10mins and supernatant completely aspirated.

The magnetic labelling of cells was performed by resuspending 10^7^cells in 80μl of MACS running buffer with the addition of 20μl of anti-CD14 human microbeads (MACS Miltenyi Biotec, (Catalogue no.130-050-201) Bergisch-Gladbach, Germany) mixing and incubating on ice for 15mins. After incubation, cells were washed with 2ml of MACS running buffer and centrifuged at 300 x *g* for 10mins. The supernatant was discarded and cells were finally re-suspended in 500μl of fresh MACS running buffer.

The magnetic separation of CD14+ monocytes was performed on a MACS LS column and magnetic separator (Miltenyi Biotec, 130-042-401). The column was placed in the MACS separator and rinsed with 3ml of MACS rinsing buffer. 500μl of cell suspension was added onto the column and washed three times with 3ml of MACS rinsing buffer and the unlabelled cell suspension was collected. The column was removed from the magnetic separator and placed into a 15ml tube and CD14+ positive fraction of cells eluted using 5ml of fresh rinsing buffer. The eluted positive cell fraction was centrifuged at 220 x *g* for 8mins and the total number of CD14+ monocytes was counted using a haemocytometer with the concentration of cells was adjusted to 2×10^6^ cells/ml.

**Isolation of NK cells from PBMC**

In co-culture experiments using NK-cells/monocytes, NK cells were isolated from negative selection of PBMCs following CD14+ selection. The numbers of cells were determined from the negative fraction of PBMC and adjusted to 10^7^ cells /100 µl of MACS running buffer. Primary labelling of cells was performed with mouse anti-bovine CD335 antibody (Clone AKS1 rat IgG1 isotype; Catalog no. MCA2365GA, AbD serotec) at concentration of 1/50 with incubation on ice for 15mins. Cells were washed with 1-2 ml of running buffer at 300 x *g* for 10mins.

Supernatant was aspirated, and 10^7^ cells were resuspended into 80µl of running buffer containing 20µl rat anti-mouse IgG1 microbeads (Catalogue no. 130-047-102 Miltenyi Biotec,) then incubated on ice for 15mins. Cells were washed with 1-2 ml of running buffer at 300 x *g* for 10 mins and resuspended into fresh 500µl of running buffer. Magnetic separation of NK-cells was performed by washing an LS column with 3ml of rinsing buffer. 500µl of cell suspension was added into the column and washed 3 times with 3 ml of rinsing buffer. Final NK-cell elution was carried out by addition of 5ml of rinsing buffer. NK-cells were counted and stimulated with recombinant bovine IL-15 (Kingfisher Biotech Inc.) at a concentration of 10ng/ml. The cells were seeded into 24 well plate (1x10^4^ cells/well) and incubated at 37°C for overnight.

**Infection of CD14+monocytes with N. caninum tachyzoites and co-culture with NK-cells**

Stimulated NK cells were collected by washing with 200µl ice cold D-PBS/well and were transferred into 1.5ml tubes and kept at 4°C until used. Purified *N. caninum* tachyzoites (10^7^/ml) were resuspended in D-PBS and fluorescent dye carboxyfluorescein succinimidyl ester (CFSE) was added at a concentration of 5µM/10^7^ parasites and incubated for 10mins at room temperature. Following incubation, 400µl of RPMI complete media was added and the parasites kept on ice for 5mins before washing at 448 x *g* for 5mins. The supernatant was aspirated and pellet resuspended in 1ml of D-PBS. 1x10^5^ purified stained *N. caninum* tachyzoites were used to infect CD14^+^ monocytes (MOI 1:10) in 24 well cell culture plates and incubated for 3hrs at 37°C. Afterward the supernatant was removed and placed with fresh RPMI complete media and 1x10^4^ washed NK-cells were added into *N. caninum* infected and uninfected wells, giving an target:effector ratio of 10:1. In control wells only CD14+ monocytes and CD14+ monocytes/tachyzoites were cultured without the addition of NK-cells.

**In-vitro culture of *N. caninum* in Vero cells**

In vitro culture of *N. caninum* tachyzoites was performed in Vero cell lines using the isolate Nc-Liv (Barber et al., 1995) was obtained from University of Liverpool. The frozen parasite was thawed in a water bath at 37°C and centrifuged at 448 x *g* for 10mins using 10ml of DMEM media supplemented with 10% FBS and 100 U/mL penicillin/ 100µg/mL streptomycin. The supernatant was discarded and parasite finally resuspended in 2ml of fresh DMEM media enumeration of tachyzoites. In-vitro culture of *N. caninum* was performed on a confluent monolayer of Vero cells with a total 9x10^5^ parasite per 25 cm2 flask used.

RNA isolation

RNA isolation was performed from infected and uninfected CD14^+^ monocytes grown in a monolayer. The monolayer of cells was washed after aspiration of media with fresh cold D-PBS to remove any residual media and lysed directly by adding of 350µl of buffer RLT in 24 well plates. Cell lysate was collected and transferred into RNAse free microcentrifuge tube. Homogenization of lysate was performed by vortex and pipetting to ensure homogenisation. An equal volume of 70% molecular grade ethanol was added to the homogenized lysate and RNA isolated using the RNeasy kit (Qiagen) according to manufacturer’s instructions.

DNase treatment

Eluted total RNA was treated with Ambion DNA free DNase kit (Catalogue no. AM1906) to eliminate genomic DNA from samples. The total volume of eluted RNA was adjusted to 45µl and 5µl of 10X buffer and 1µl of rDNase was added and incubated the samples at 37°C for 30 mins. DNase activity was inactivated by adding of 6µl of DNase inactivation reagent at the end of incubation period, with treated RNA collected after centrifugation 10,000 x *g*, 2mins

Quality analysis

The quality and quantity of RNA samples was evaluated by using Nanodrop. Quality assessment of RNA was performed via absorbance 260/280 ratio with 1.7-2.1 accepted as good quality.

Microarray preparation, hybridisation and scanning

Duplicate total RNA samples from young (n=3) and adult monocytes (n=2) were used for Agilent One-Color Microarray-based Gene Expression Analysis using cyanine 3-labeled targets to measure gene expression in *N. caninum* infected and non-infected control samples. The Bovine (V2) Gene Expression Microarray, 4×44K chip (G2519F-023647) was used in this experiment. cDNA samples were prepared from total RNA samples of infected and control cattle monocytes using Affinity Script RT oligo dT promoter primers by reverse transcription and then RNA was generated from cDNA via transcription using T7 RNA polymerase and cyanine 3 CTP. The Cyanine 3-labeled, linear amplified cRNA was hybridised onto microarray slides and washed to remove unbound RNA from the slides. The slides were scanned using an Agilent C Scanner for feature extraction.

Scanned microarray images were extracted in GeneSpring GX 9.0. Data text files were converted into Excel files and background mean signals were subtracted. The raw data were analysed by uploading the mean files into network analyst a web based tool for gene expression profiling (1). Data were normalised by a log2 transformation and low variance genes were filtered on base of interquartile range (IQR) for equal difference among upper and lower quartile. The differential gene expression was identified by limma method by using adjusted P value <0.05 and fold change >2.0 (2). Specific comparisons were performed between infected and control group of neonatal and adult cattle to determine the set of differentially expressed genes. The interaction across neonatal and adult cattle was examined via nested comparison in infected and control groups.

Evaluation of genes of interest

An evaluation of genes of interest was performed among neonates and adult infected versus uninfected monocytes in duplicate. The mean signal was obtained after subtracting background signals from each replicate with corresponding genes evaluated using the R program (1).

Initially, 44,469 genes were analysed between infected neonate versus uninfected and infected adult versus uninfected. 25,642 annotated genes were matched and used to determine any differentially expressed genes in each age group after removal of duplicate genes IDs. To determine specific interaction between adult infected versus neonate infected and adult uninfected versus neonate uninfected, a nested comparison was performed.

Protein-protein interaction analysis was performed manually for identification of different pathways that were associated with changes in particular biological processes. Additionally, a full list of differentially expressed genes was reviewed for those known to be involved in age related immune responses and functions of various immune pathways related genes across neonatal and adult cattle.

# Supplementary Figures and Tables

## Supplementary Figures


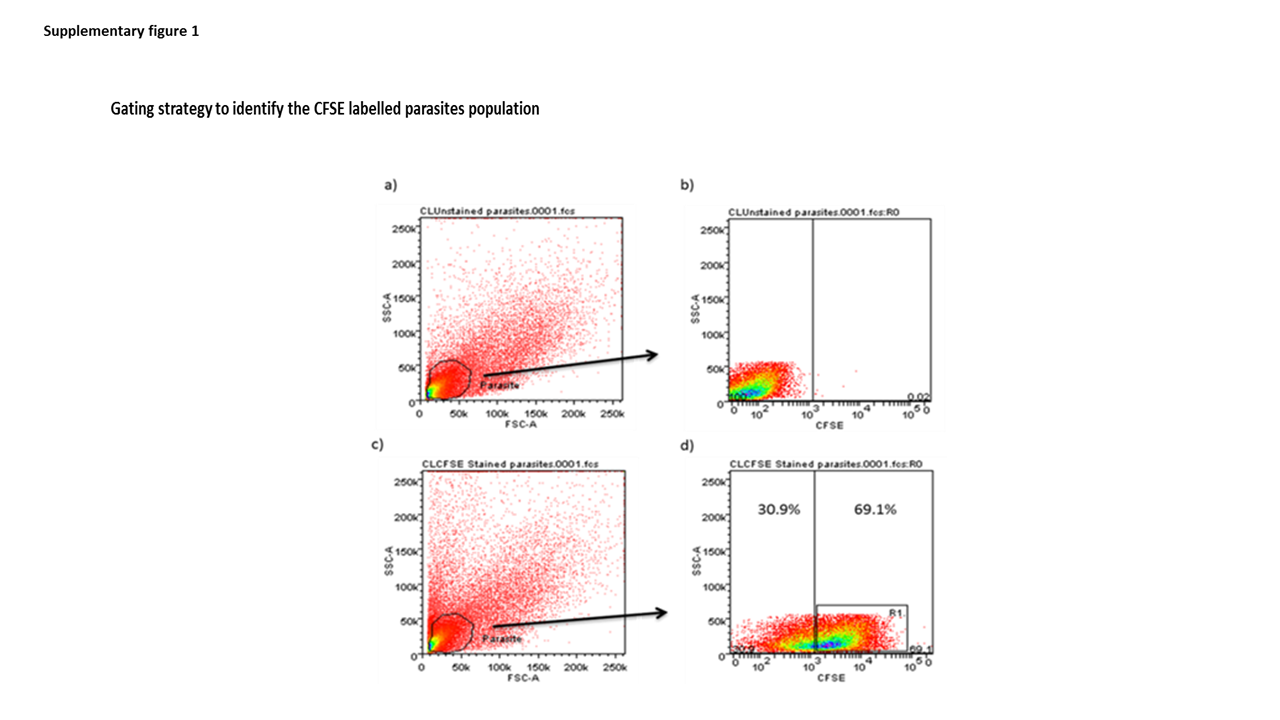


Supplementary Figure 1. Gating strategy to determine the percent of CFSE labelled parasites

**(a)** A parasite population was gated on the basis of forward and side scatter dot plot of unstained parasites. **(b)** Gated unstained parasite population showing no CFSE staining. **(c)** CFSE labelled parasite population were gated based on forward and side scatter dot plot. **(d)** Gated stained parasite population showing CFSE labelled parasites (R1) population (69.1%).


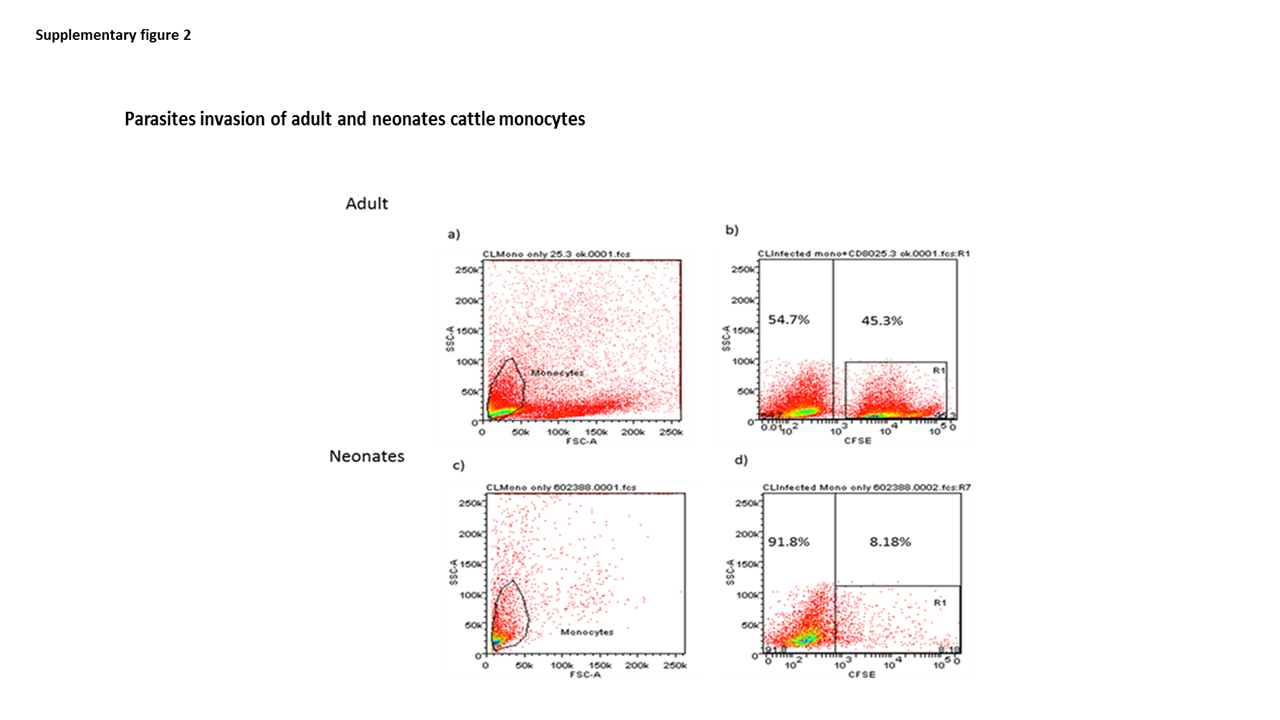


Supplementary Figure 2. Gating strategy to identify the infected CD14^+^ monocytes population of adult and neonatal cattle

(a & c) Unstained CD14+ monocytes of adult and neonates were gated on the basis of forward and side scatter dot plot respectively. (b & d) CFSE positive infected CD14+monocytes were identified within gated population of monocytes of adult and neonates. These dot plots are representative data from one adult and neonates cattle among 6 adult cattle and 4 neonatal calves respectively.


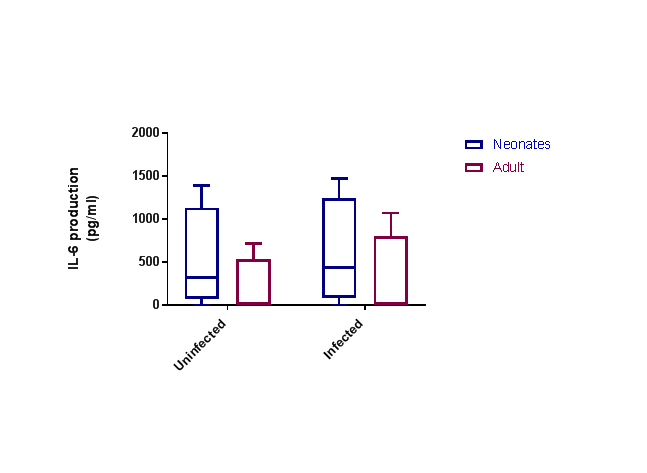


**Supplementary Figure 3.** **IL-6 production from *N. caninum* infected and uninfected CD14^+^ monocyte of neonates and adult cattle**

IL-6 production was determined from *N. caninum* infected and uninfected CD14^+^ monocytes of neonates and adult cattle after 24hrs post infection, supernatants were collected and analysed by ELISA in duplicate. These data showing median with 95% confidence interval of neonates (n=4) and adult cattle (n=4). Statistical analysis was performed by using a Mann Whitney test.

Table S1. Significantly upregulated biological pathways in neonates infected versus uninfected

| **Pathway** | **Total no. of genes** | **No. of Upregulated genes** | ***P Value*** |
| --- | --- | --- | --- |
| nucleosome assembly | 233 | 97 | 4.16E-28 |
| hemopoiesis | 53 | 34 | 5.39E-18 |
| base-excision repair | 68 | 36 | 3.34E-15 |
| regulation of secretion | 137 | 54 | 6.04E-15 |
| positive regulation of transferase activity | 166 | 59 | 7.76E-14 |
| Rho protein signal transduction | 94 | 41 | 2.07E-13 |
| DNA-dependent transcription, elongation | 106 | 44 | 2.31E-13 |
| DNA catabolic process | 16 | 15 | 6.91E-13 |
| DNA damage checkpoint | 487 | 117 | 9.42E-12 |
| nitrogen compound metabolic process | 227 | 68 | 1.06E-11 |
| alcohol metabolic process | 87 | 36 | 3.91E-11 |
| regulation of transcription from RNA polymerase II promoter | 498 | 117 | 4.37E-11 |
| energy reserve metabolic process | 206 | 62 | 7.03E-11 |
| hormone secretion | 81 | 34 | 8.53E-11 |
| DNA damage response, signal transduction by p53 class mediator | 317 | 83 | 1.30E-10 |
| positive regulation of cell adhesion | 120 | 43 | 1.40E-10 |
| DNA replication initiation | 604 | 133 | 2.22E-10 |
| organic acid metabolic process | 13 | 12 | 2.61E-10 |
| JAK-STAT cascade | 291 | 77 | 3.62E-10 |
| steroid metabolic process | 349 | 87 | 6.96E-10 |
| intrinsic apoptotic signaling pathway | 36 | 20 | 1.58E-09 |
| tRNA metabolic process | 27 | 17 | 1.89E-09 |
| lipid biosynthetic process | 374 | 90 | 2.40E-09 |
| phagocytosis | 103 | 37 | 2.48E-09 |
| regulation of cytokine biosynthetic process | 708 | 146 | 3.48E-09 |
| epithelial cell differentiation | 34 | 19 | 3.57E-09 |
| regulation of protein metabolic process | 147 | 44 | 4.90E-08 |
| viral reproduction | 17 | 12 | 7.48E-08 |
| actin filament-based process | 161 | 45 | 3.17E-07 |
| protein polymerization | 54 | 22 | 3.44E-07 |
| cell maturation | 426 | 92 | 3.90E-07 |
| aerobic respiration | 72 | 26 | 5.15E-07 |
| response to hypoxia | 105 | 33 | 6.69E-07 |
| actin filament organization | 30 | 15 | 1.11E-06 |
| regulation of MAPK cascade | 93 | 30 | 1.15E-06 |
| homeostasis of number of cells | 406 | 86 | 2.18E-06 |
| DNA-dependent DNA replication | 68 | 24 | 2.25E-06 |
| post-Golgi vesicle-mediated transport | 18 | 11 | 2.32E-06 |
| tyrosine phosphorylation of STAT protein | 232 | 55 | 5.15E-06 |
| cell-cell adhesion | 49 | 19 | 5.20E-06 |
| apoptotic DNA fragmentation | 62 | 22 | 5.35E-06 |
| transcription, DNA-dependent | 45 | 18 | 5.47E-06 |
| pyrimidine nucleotide metabolic process | 58 | 21 | 6.05E-06 |
| DNA repair | 338 | 73 | 6.25E-06 |
| positive regulation of cellular component organization | 11 | 8 | 9.28E-06 |
| double-strand break repair | 27 | 13 | 9.96E-06 |
| receptor-mediated endocytosis | 51 | 19 | 1.03E-05 |
| carbohydrate transport | 146 | 38 | 1.60E-05 |
| positive regulation of transcription, DNA-dependent | 28 | 13 | 1.64E-05 |
| stress-activated protein kinase signaling cascade | 66 | 22 | 1.69E-05 |
| mitotic sister chromatid segregation | 25 | 12 | 2.29E-05 |
| neutral amino acid transport | 12 | 8 | 2.47E-05 |
| mRNA processing | 19 | 10 | 4.10E-05 |
| nucleotide-excision repair | 89 | 26 | 4.20E-05 |
| positive regulation of T cell proliferation | 126 | 33 | 4.95E-05 |
| humoral immune response | 48 | 17 | 6.37E-05 |
| phosphatidylinositol biosynthetic process | 270 | 58 | 6.47E-05 |
| multicellular organismal development | 44 | 16 | 7.23E-05 |
| apoptotic nuclear changes | 178 | 42 | 7.32E-05 |
| chromatin remodeling | 49 | 17 | 8.59E-05 |
| embryo implantation | 36 | 14 | 8.73E-05 |
| immune response | 28 | 12 | 9.20E-05 |
| cation transport | 17 | 9 | 9.53E-05 |
| cellular aromatic compound metabolic process | 8 | 6 | 0.000107 |
| response to drug | 8 | 6 | 0.000107 |
| tissue remodeling | 41 | 15 | 0.000112 |
| RNA catabolic process | 18 | 9 | 0.000169 |
| cytoskeleton organization | 214 | 47 | 0.00018 |
| fatty acid oxidation | 292 | 60 | 0.00018 |
| DNA-dependent transcription, initiation | 22 | 10 | 0.000198 |
| one-carbon metabolic process | 22 | 10 | 0.000198 |
| steroid biosynthetic process | 215 | 47 | 0.000202 |
| M phase of mitotic cell cycle | 57 | 18 | 0.000212 |
| negative regulation of transcription from RNA polymerase II promoter | 15 | 8 | 0.000223 |
| muscle organ development | 15 | 8 | 0.000223 |
| regulation of endocytosis | 15 | 8 | 0.000223 |
| positive regulation of cell differentiation | 35 | 13 | 0.000267 |
| regulation of pH | 12 | 7 | 0.000272 |
| apoptotic signaling pathway | 63 | 19 | 0.00028 |
| chromosome condensation | 9 | 6 | 0.000285 |
| regulation of small GTPase mediated signal transduction | 4 | 4 | 0.000286 |
| positive regulation of NF-kappaB transcription factor activity | 23 | 10 | 0.00031 |
| small GTPase mediated signal transduction | 36 | 13 | 0.000369 |
| reciprocal meiotic recombination | 145 | 34 | 0.000389 |
| rhythmic process | 28 | 11 | 0.000451 |
| regulation of body fluid levels | 24 | 10 | 0.000469 |
| regulation of DNA binding | 47 | 15 | 0.000619 |
| regulation of cyclin-dependent protein kinase activity | 52 | 16 | 0.00065 |
| lysosomal transport | 62 | 18 | 0.000668 |
| regulation of cell growth | 43 | 14 | 0.000746 |
| androgen receptor signaling pathway | 54 | 16 | 0.00103 |
| MAPK cascade | 22 | 9 | 0.00106 |
| meiotic cell cycle | 65 | 18 | 0.00123 |
| cell migration | 5 | 4 | 0.00128 |
| positive regulation of DNA binding | 5 | 4 | 0.00128 |
| regulation of cell differentiation | 5 | 4 | 0.00128 |
| macromolecule biosynthetic process | 252 | 50 | 0.00136 |
| inorganic anion transport | 115 | 27 | 0.00145 |
| vesicle-mediated transport | 8 | 5 | 0.00148 |
| cellular homeostasis | 23 | 9 | 0.00154 |
| protein processing | 46 | 14 | 0.00157 |
| RNA 3'-end processing | 46 | 14 | 0.00157 |
| regulation of Rho protein signal transduction | 28 | 10 | 0.00192 |
| positive regulation of cysteine-type endopeptidase activity involved in apoptotic process | 42 | 13 | 0.00192 |
| cell cycle checkpoint | 3 | 3 | 0.0022 |
| response to toxin | 3 | 3 | 0.0022 |
| anion transport | 53 | 15 | 0.00242 |
| anatomical structure morphogenesis | 91 | 22 | 0.00261 |
| positive regulation of binding | 169 | 35 | 0.00327 |
| isoprenoid metabolic process | 60 | 16 | 0.00342 |
| protein modification process | 21 | 8 | 0.00342 |
| DNA metabolic process | 6 | 4 | 0.00345 |
| lipoprotein metabolic process | 13 | 6 | 0.00365 |
| G2/M transition of mitotic cell cycle | 77 | 19 | 0.00389 |
| positive regulation of cytokine secretion | 1160 | 181 | 0.00397 |
| establishment of organelle localization | 31 | 10 | 0.00451 |
| ribosome biogenesis | 22 | 8 | 0.00477 |
| positive regulation of protein metabolic process | 10 | 5 | 0.00531 |
| protein localization | 32 | 10 | 0.0058 |
| RNA splicing, via transesterification reactions | 121 | 26 | 0.00631 |

Table S2. Significantly upregulated biological pathways in adult infected versus uninfected.

| **Pathway** | **Total no. of genes** | **No. of Upregulated genes** | ***P value*** |
| --- | --- | --- | --- |
| nucleosome assembly | 233 | 68 | 1.29E-80 |
| JAK-STAT cascade | 291 | 21 | 4.08E-11 |
| intrinsic apoptotic signaling pathway | 36 | 8 | 8.75E-09 |
| regulation of endocytosis | 15 | 6 | 1.32E-08 |
| hormone secretion | 81 | 10 | 4.51E-08 |
| positive regulation of hydrolase activity | 59 | 8 | 5.07E-07 |
| actin filament organization | 30 | 6 | 1.34E-06 |
| response to drug | 8 | 4 | 1.4E-06 |
| base-excision repair | 68 | 8 | 1.54E-06 |
| epithelial cell differentiation | 34 | 6 | 2.92E-06 |
| gene silencing | 13 | 4 | 1.37E-05 |
| immune response | 28 | 5 | 1.93E-05 |
| hemopoiesis | 53 | 6 | 4.13E-05 |
| positive regulation of NF-kappaB transcription factor activity | 23 | 4 | 0.000154 |
| response to hypoxia | 105 | 7 | 0.000283 |
| inorganic anion transport | 115 | 7 | 0.000492 |
| tyrosine phosphorylation of STAT protein | 232 | 10 | 0.000522 |
| positive regulation of transferase activity | 166 | 8 | 0.00093 |
| stress-activated protein kinase signaling cascade | 66 | 5 | 0.00121 |
| apoptotic process | 20 | 3 | 0.00172 |
| cell projection assembly | 6 | 2 | 0.00213 |
| DNA-dependent transcription, initiation | 22 | 3 | 0.00228 |
| chromatin remodeling | 49 | 4 | 0.00289 |
| actin filament-based process | 161 | 7 | 0.00346 |
| cell-matrix adhesion | 8 | 2 | 0.00391 |
| chromosome condensation | 9 | 2 | 0.00499 |
| interleukin-2 production | 9 | 2 | 0.00499 |
| cell maturation | 426 | 12 | 0.00565 |
| apoptotic signaling pathway | 63 | 4 | 0.00714 |
| small GTPase mediated signal transduction | 36 | 3 | 0.00935 |
| negative regulation of apoptotic process | 37 | 3 | 0.0101 |
| proteoglycan biosynthetic process | 70 | 4 | 0.0103 |
| negative regulation of transcription, DNA-dependent | 1 | 1 | 0.0122 |
| DNA damage response, signal transduction by p53 class mediator | 317 | 9 | 0.0152 |
| regulation of cytokine production | 16 | 2 | 0.0157 |
| RNA splicing, via transesterification reactions | 121 | 5 | 0.0159 |
| lipid catabolic process | 17 | 2 | 0.0177 |
| DNA repair | 338 | 9 | 0.022 |
| sexual reproduction | 2 | 1 | 0.0242 |
| regulation of cyclin-dependent protein kinase activity | 52 | 3 | 0.0252 |
| protein modification process | 21 | 2 | 0.0265 |
| actin polymerization or depolymerization | 140 | 5 | 0.0279 |
| embryonic morphogenesis | 22 | 2 | 0.0289 |
| cellular homeostasis | 23 | 2 | 0.0314 |
| cytoskeleton-dependent intracellular transport | 23 | 2 | 0.0314 |
| regulation of body fluid levels | 24 | 2 | 0.034 |
| synaptic transmission | 59 | 3 | 0.0348 |
| DNA damage checkpoint | 487 | 11 | 0.0348 |
| mitotic sister chromatid segregation | 25 | 2 | 0.0366 |
| lysosomal transport | 62 | 3 | 0.0395 |
| meiotic cell cycle | 65 | 3 | 0.0444 |
| regulation of Rho protein signal transduction | 28 | 2 | 0.0451 |
| interleukin-8 biosynthetic process | 29 | 2 | 0.0481 |

**References**

1. Xia J, Gill EE, & Hancock RE (2015) NetworkAnalyst for statistical, visual and network-based meta-analysis of gene expression data. *Nat Protoc* 10(6):823-844.

2. Mariani TJ*, et al.* (2003) A variable fold change threshold determines significance for expression microarrays. *FASEB J* 17(2):321-323.
